# A mixed methods study to evaluate the clinical and cost-effectiveness of a self-managed exercise programme versus usual physiotherapy for chronic rotator cuff disorders: protocol for the SELF study

**DOI:** 10.1186/1471-2474-13-62

**Published:** 2012-04-30

**Authors:** Chris Littlewood, Jon Ashton, Sue Mawson, Stephen May, Stephen Walters

**Affiliations:** 1 School of Health & Related Research, University of Sheffield, Regent Court, 30 Regent Street, Sheffield, S1 4DA, UK; 2 Physiotherapy Services, Doncaster Royal Infirmary, Armthorpe Road, Doncaster, DN2 5LT, UK; 3 Director of the National Institute for Health Research (NIHR) Collaborations for Leadership in Applied Health Research & Care (CLAHRC) for South Yorkshire, Sheffield Teaching Hospitals, 11 Broomfield Road, Sheffield, S10 2SE, UK; 4 Faculty of Health & Wellbeing, Sheffield Hallam University, Sheffield, S10 2BP, UK

**Keywords:** Mixed methods study, Randomised controlled trial, Rotator cuff tendinopathy, Exercise, Rehabilitation, Quality of life

## Abstract

**Background:**

Shoulder pain is the third most common reason for consultation with a physiotherapist and up to 26% of the general population might be expected to experience an episode at any one time. Disorders of the shoulder muscles and tendons (rotator cuff) are thought to be the commonest cause of this pain. The long-term outcome is frequently poor despite treatment. This means that many patients are exposed to more invasive treatment, e.g. surgery, and/or long-term pain and disability.

Patients with this disorder typically receive a course of physiotherapy which might include a range of treatments. Specifically the value of exercise against gravity or resistance (loaded exercise) in the treatment of tendon disorders is promising but appears to be under-used. Loaded exercise in other areas of the body has been favourably evaluated but further investigation is needed to evaluate the impact of these exercises in the shoulder and particularly the role of home based or supervised exercise versus usual treatment requiring clinic attendance.

**Methods/Design:**

A single-centre pragmatic unblinded parallel group randomised controlled trial will evaluate the effectiveness of a self-managed loaded exercise programme versus usual clinic based physiotherapy. A total of 210 study participants with a primary complaint of shoulder pain suggestive of a rotator cuff disorder will be recruited from NHS physiotherapy waiting lists and allocated to receive a programme of self-managed exercise or usual physiotherapy using a process of block randomisation with sealed opaque envelopes. Baseline assessment for shoulder pain, function and quality of life will be undertaken with the Shoulder Pain & Disability Index, the Patient Specific Functional Scale and the SF-36. Follow-up evaluations will be completed at 3, 6 and 12 months by postal questionnaire. Both interventions will be delivered by NHS Physiotherapist’s.

An economic analysis will be conducted from an NHS and Personal Social Services perspective to evaluate cost-effectiveness and a qualitative investigation will be undertaken to develop greater understanding of the experience of undertaking or prescribing exercise as a self-managed therapy.

**Trial registration number:**

ISRCTN84709751

## Background

Shoulder pain is one of the most common musculoskeletal symptoms with prevalence estimated at between 16 to 26% in the general population at any one time [1,2]. It is the third most common primary care musculoskeletal presentation [3] and the third most common reason for consultation with a physiotherapist [4]. Impaired shoulder function impacts significantly upon activities of daily living, including eating, dressing and working [5]. Disorders of the shoulder muscles and tendons (rotator cuff) are the commonest cause of this pain [6]. However long-term outcome is frequently poor for a significant proportion of patients which means that many are subsequently exposed to more invasive and costly treatment options, e.g. injections, surgery, and/or long-term pain and disability [7].

Shoulder pain, incorporating rotator cuff disorders, is a significant burden to the NHS and society. It has been identified that around 1% of adults in the UK consult their GP with a new presentation of shoulder pain each year. This amounts to over 500,000 adults. Costs in the first 6 months following primary care contact have been estimated to be €690 which means that costs attributable to this problem are in the region of €345 million or £310 million per year. Almost 50% of this cost is attributable to sick leave from paid employment [8]. Additionally, in 2005/6, 16,885 patients were admitted to hospital diagnosed with problems relating to the rotator cuff [9]. NHS costs associated with surgical procedures for such shoulder problems are estimated at £1,762 [10], which equates to a conservative estimate of almost £30 million per year. Clearly chronic rotator cuff disorders bring the associated health costs and economic burden, including loss of productivity, associated with other chronic conditions [5]. Therefore, this is an important health and social care problem for patients, clinicians, commissioners and researchers to consider.

Despite rotator cuff disorders being such a common shoulder problem there is a lack of high quality studies upon which to base practice [11]. Numerous systematic reviews have been undertaken in relation to subacromial impingement syndrome, an umbrella term encompassing rotator cuff disorders, investigating the various plausible interventions including physiotherapy, corticosteroid injections and surgery but all identify the insufficiency of the evidence base when attempting to draw conclusions [12-17]. Specifically in relation to the rotator cuff, a recent systematic review, which included 4 randomised controlled trials, suggested that loaded exercise, i.e. exercise against gravity or resistance, in the treatment of this disorder was promising [18] but due to the paucity of research and methodological limitations of the included studies further research is warranted. It was also recognised that home based or supervised exercise appears to confer similar outcomes to interventions, including therapist-led interventions, which were offered in a clinic-based setting. Additionally the benefits of loaded exercise have been reported in a particularly resistant patient group with long standing shoulder pain awaiting surgery due to a lack of response to previous conservative care [19]. Although small (n = 9), this uncontrolled pilot study reported that 56% responded to the point where they no longer required surgery despite a mean duration of pain of 41 months. Chronic rotator cuff tendon disorders have been shown to demonstrate similar pathological changes to tendon disorders in other areas of the body, e.g. the elbow, Achilles tendon, patellar tendon [20] where favourable results including reduced pain and improved function have been demonstrated in response to similar loaded exercise [21].

A recent survey of current practice suggests that physiotherapists usually offer a wide range of interventions for rotator cuff disorders including advice, stretching, exercise, manual therapy, massage, strapping, acupuncture, electrotherapy, corticosteroid injection [22]. The majority of UK physiotherapists would expect patients to engage with some level of self-management but more than 58% would ask the patient to return for therapist-led interventions. The variability in the approach of physiotherapists might reflect the lack of high quality evidence upon which practice can be based.

It is hypothesised that self-managed loaded exercise has the capacity to reduce costs associated with rotator cuff disorders and improve treatment outcome which therefore reduces the need for work absenteeism and further expensive invasive interventions. There is a clear need for high quality research in this area to inform clinical practice and commissioning priorities. At a time when healthcare agendas are emphasising the need to encourage the self-management of long-term conditions this research would build upon the developmental work that has been undertaken so far and, based upon high quality research methods, will offer useful clinical and cost data upon which practice can be developed and future research can be based.

## Methods/Design

### Aims

The proposed study aims to answer the question: Is a self-managed exercise programme more effective than usual physiotherapy for chronic rotator cuff disorders?

The objectives are:

· To evaluate clinical effectiveness of loaded exercise in terms of pain, function and well-being.

· To evaluate cost-effectiveness of the interventions.

· To investigate patient adherence with treatment.

· To explore the perceptions and experience of the study participants.

### Methods

The study design is a single-centre pragmatic unblinded parallel group randomised controlled trial (RCT) combined with qualitative investigation of patient and therapist experience. The study will be based at Doncaster & Bassetlaw Hospitals NHS Foundation Trust, UK.

The first part of the proposed study will be a RCT comparing self-managed loaded exercise versus usual physiotherapy.

### The intervention

The intervention is self-managed loaded (against gravity or resistance) exercise. The exercise, prescribed by the physiotherapist but completed by the patient independently, involves exercising the affected shoulder against gravity, a resistive therapeutic band or hand weight over 3 sets of 10 to 15 repetitions completed twice per day. This exercise can be uncomfortable for the patient but is prescribed to ensure that the discomfort is manageable. Exercise prescription is guided by symptomatic response requiring that pain is produced during exercise that remains no worse upon cessation of that exercise [7,23]. Hence participants with more severe symptoms tend to commence a lighter regime to begin with. A typical programme is presented in Box 1.

**Box 1:** Typical loaded exercise progression

1. Week 0: Baseline assessment:
·Resisted isometric (no movement) shoulder abduction (taking the arm out to the side) against a wall, or
·Resisted shoulder abduction from 0 to 30° using moderate resistance from Theraband (resistive band used for training purposes).
2. Week 3: Initial follow-up:
·Resisted shoulder abduction from 80 to 120° using light weight, e.g. tin of food.
3. Week 6: Second follow-up:
·Resisted shoulder abduction from 80 to 120° with progressively increasing repetition and weight, e.g. heavy Theraband or dumbbell.
4. Week 12: Final follow-up/discharge.

To maintain the pragmatic value of the study, in both arms of the trial, the treating physiotherapists will determine the number of sessions, frequency and point of treatment cessation. However, the emphasis of the intervention arm is towards self-management with supervision and guidance only offered by the physiotherapist [7,23].

### The comparator

Usual physiotherapy might include a range of interventions including advice, stretching, exercise, manual therapy, massage, strapping, acupuncture, electrotherapy, corticosteroid injection at the discretion of the treating physiotherapist.

Prior to the recruitment of patients into the trial, the physiotherapists of Doncaster & Bassetlaw Hospitals NHS Foundation Trust who usually treat patients with rotator cuff disorders will be invited to participate in the study. A participant information sheet will be provided and explained via an initial information session delivered by the research team. If they wish to be included they will be asked to complete a consent form and return to the chief investigator within 2 weeks. During these training sessions the issue of contamination of the control arm and the implications will be discussed in an attempt to minimise contamination. The degree of any contamination will be evaluated via review of patient records and reported accordingly.

### Recruitment

Inclusion criteria will be: (i) Age ≫ 18 years, (ii) Willing and able to participate, (iii) Primary complaint of shoulder pain with or without referral into the upper limb for ≫ 3months, (iv) No/minimal resting shoulder pain, (v) Range of shoulder movement largely preserved, and (vi) Shoulder pain provoked consistently with resisted muscle tests, usually abduction or lateral rotation.

Exclusion criteria will be: (i) Shoulder surgery within last 6 months, (ii) Reasons to suspect systemic pathology including inflammatory disorders, (iii) Cervical repeated movement testing affects shoulder pain and/or range of movement. People who are unable to understand written and spoken English will be included in the study and NHS translation services will be accessed to accommodate their needs.

Potential trial participants will be identified from the NHS physiotherapy waiting list by a physiotherapist who usually has access to this information as part of their clinical role. Initial contact will be made through an introductory letter. Along with this letter the potential participants will also receive a participant information sheet and consent form. The letter will be followed up with a telephone call made by the physiotherapist one week later where further study information will be relayed as required and an enquiry about further participant involvement will be made. If the call recipient expresses interest in participating in the study the physiotherapist will then undertake initial telephone screening for inclusion criteria i to iv and exclusion criteria i to ii. If these criteria are met then the potential participant will be invited to read the participant information sheet, ask any questions and sign the consent form prior to attending for physical examination screening for inclusion criteria v to vi and exclusion criteria iii. If the participant does not wish to pursue the discussion or does not meet the criteria they will be thanked for their time and told that their referral will continue to be treated as per usual arrangements.

Physical examination screening will also be carried out by an experienced clinical physiotherapist. The physical examination screening will take up to 30 minutes and will include assessment of neck and shoulder movements and any associated symptomatic responses as per a typical musculoskeletal examination. If potential participants do not meet inclusion criteria v to vi they will be thanked for their time, offered advice about their presenting problem in line with the physical examination screening, e.g. advice to keep the arm moving, offered a generic advice leaflet produced by the Physiotherapy department at the Doncaster & Bassetlaw Hospitals NHS Foundation Trust and told that their referral will continue to be treated as per usual arrangements. This research study will not interfere with the timing of receiving physiotherapy. The process of including or excluding participants will take place whilst the referral remains on the waiting list.

### Informed consent

Prior to attending the physical examination screening a participant information sheet and consent form will be provided by post. The physiotherapist will offer an overview of the information sheet and answer any questions at the time of the initial telephone call. The potential participants will then be offered an appointment for a physical examination screening or, if more time is needed, will be offered a follow-up telephone call within the proceeding 2 weeks. If the participant does not wish to be considered for the study they will be thanked for their time and told that their referral will continue to be treated as per usual arrangements.

The potential participants will provide a signed consent form prior to the physical examination screening tests. Where consent is gained, the participants General Practitioner will also be informed of their inclusion in the study by letter. All potential participants who meet the criteria following physical examination screening will be eligible to be randomised. Those participants who do not meet the criteria will not be eligible to be randomised and will be informed of this along with the reasons. We expect only a small minority to be excluded at this stage and this aspect of the process is made clear in the information sheet.

### Baseline/outcome assessment

After the participants have been assessed for eligibility and consent has been gained, prior to randomisation, they will complete a range of appropriate patient-reported outcome measures to establish baseline pain, function, quality of life and level of self-efficacy. Additionally, the participants’ preference for one treatment or the other, if they had a free choice, will be noted to enable analysis of the effect of preference on outcome.

The primary aim of the proposed study is to evaluate the clinical and cost-effectiveness of self-managed loaded exercise versus usual physiotherapy for rotator cuff disorders. The primary outcome measure will be the Shoulder Pain & Disability Index (SPADI) [24] score at 12 weeks. SPADI is a self-report measure specifically developed to evaluate pain and function in patients with shoulder pathology [25]. It is a commonly used measure which has been validated for use in this patient population and a minimally clinically important change of 10 points has been identified [25]. The SPADI includes 13 items divided into 2 sub-scales; pain (5 items), disability (8 items). The responses are indicated on a visual analogue scale where 0 = no pain/no difficulty and 10 = worst imaginable pain/so difficult it requires help. The items are summed and converted to a total score out of 100.

The Short-form 36 (SF-36) is a generic measure of health related quality of life [26] and is the most widely used measure of this nature. The SPADI & SF-36 will be repeated at 12, 24 and 52 weeks and returned by post. These measures will be complemented by the patient specific functional scale (PSFS) which is a patient-specific outcome measure which investigates functional status and is intended to complement the findings of generic or condition-specific measures [27]. The PSFS has been shown to be valid and responsive in various musculoskeletal populations [28]. Due to the nature of the PSFS, this measure will be completed at baseline and then completed, until the end of the treatment episode, in the presence of the attending physiotherapist. Treatment duration is likely to be in the region of 3 months with the PSFS being completed at 1, 2 and 3 months post baseline.

We are interested in evaluating levels of adherence with treatment but also exploring possible factors that might predict non-adherence in this context. A range of such factors have been identified including level of pain at baseline, levels of physical functioning, levels of well-being [29], all of which can be captured with the aforementioned measures. However, levels of self-efficacy appear to be an important determinant of adherence [29] and so this will be captured via the General Self-efficacy scale (GSES) [30] at baseline. The GSES is a 10-item measure that has been developed to measure this construct and has been validated across different populations in different countries [31].

Due to the widely recognised limitations of clinical measures including range of movement and strength [32] these measures will not be undertaken as part of this study. In the absence of objectives measures of adherence, levels of treatment adherence will be formally measured through the use of an exercise diary including number and percentage of treatment sessions attended and percentage of exercises completed as reported by the patient.

### Randomisation

Block randomisation will be carried out by an administrator based in the physiotherapy department but independent to the study. Following receipt of written informed consent and baseline assessment, a computer generated random number sequence indicating group allocation will be concealed in sealed opaque envelopes which will be consecutively numbered. The group allocation and baseline PSFS assessment will be attached to the patient referral for the attention of the treating physiotherapist in readiness for the programme of treatment.

### Sample size calculation

The sample size calculation is based upon the primary outcome measure, the SPADI, where a 10-point change is regarded as a minimally clinically important change in shoulder function [24]. Assuming a standard deviation of 24 points, a power of 80% and a (two-sided) significance level of 5%, 91 participants per group will be required. We will allow for a 15% loss to follow-up and aim to recruit 105 participants per group. Data from the Physiotherapy department at Doncaster & Bassetlaw Hospitals NHS Foundation Trust indicates that 70 potentially suitable patients are referred for treatment each month. Hence a recruitment rate of 17 to 18 per month is felt to be realistic and manageable in the allotted timescale.

### Data analysis

As the trial is a pragmatic parallel group RCT data will be reported and presented according to the revised CONSORT statement and statistical analyses will be performed on an intention-to-treat basis. All statistical exploratory tests will be two-tailed with α = 0.05. Baseline demographic and health-related quality of life data (SF-36) will be assessed for comparability between the treatment groups. The primary aim is to compare the effect of loaded exercise versus usual physiotherapy. The mean SPADI total score at 12 weeks follow-up is the primary efficacy response variable. A two independent samples t-test will be used to compare mean SPADI total scores between the groups (loaded exercise and usual physiotherapy groups). In the event of differences between the groups with respect to baseline measurements, multiple regression or analysis of covariance (ANCOVA) will be used to adjust the treatment effect for these variables. Secondary outcomes will be analysed in a similar way. For the repeated PSFS assessments at baseline and termination of treatment and (SPADI, SF-36) assessments at baseline, 12, 24 and 52 weeks a summary measure such as the Area Under the Curve (AUC) will be calculated for each patient. A two independent samples t-test will be used to compare mean AUC between the groups (loaded exercise and usual physiotherapy groups).

### Economic analysis

A cost-utility analysis will be undertaken using a NHS and Personal Social Services (PSS) perspective. The health outcomes will be expressed in terms of quality adjusted life years (QALYs). The QALY combines length and quality of life into a single index number between 0 and 1 where 0 corresponds to a health state judged to be equivalent to death and 1 corresponds to optimal health. The SF-6D will be used to calculate QALYs. The SF-6D is composed of six multi-level dimensions which describes 18,000 health states in all. The SF-6D will be derived from a selection of SF-36 items which will be completed at baseline and follow-up visits during the trial. Any patient who completes the SF-36 can be uniquely classified according to the SF-6D.

Data regarding resource utilisation will be collected via patient notes and patient questionnaire returned at 3, 6 and 12 months along with the other measures of clinical outcome. A range of costs will be considered including the number of physiotherapy sessions attended, medications (including steroid injections) and referrals to surgery (with associated follow-up). These and other unit costs will be taken from the most recent National Reference Costs, British National Formulary and PSSRU publication ‘Unit costs of health and social care’. This will enable an estimation of the total cost for each participant as well as the average total cost for each treatment group.

As with the clinical outcomes, economic analysis will be on an intention-to-treat basis. The between groups differences will be compared using 2-independent samples t-tests. The QALY value will be estimated using straight line interpolation between data points. So, for example, if a patient reports quality of life (QoL) equal to 1 at each time point during the trial this will equate to a QALY of 1 and if they report QoL equal to 0.5 at each time point during the trial this will equate to a QALY of 0.5. Mean incremental costs and QALYs will be combined into an incremental cost effectiveness ratio (ICER) to enable assessment of the relative cost-utility. Sampling uncertainty will be represented by plots on the cost-effectiveness plane and associated cost-effectiveness acceptability curves (CEACs). To reflect uncertainty and to enable valid inferences to be made, any missing data will be imputed using multiple imputation. This process will enable a decision about the acceptability of the intervention in terms of an effective use of NHS resources.

### The qualitative investigation

The primary objective of the qualitative investigation is to explore perceptions and experience of the study participants. The intervention is largely self-managed and can be uncomfortable for patients which is in contrast to the majority view of usual physiotherapy [22]. Hence this aspect of the study is important to provide complementary data to the findings of the RCT because these factors might serve as barriers to successful outcome and/or real world implementation.

### Recruitment

Participants for the qualitative aspect of the study will be purposively selected from the treating physiotherapists and those patients recruited to the intervention arm of the RCT. All patient participants randomised to the intervention arm of the RCT will be eligible to enter and will be selected to gain a balanced sample of male/female and treatment adherers/non-adherers. All therapists involved in the delivery of the intervention will be eligible to enter.

Information relating to this aspect of the study will be included in the initial participant information sheet. The chief investigator (CL) will identify potential patient participants from the data generated by the RCT and will initially contact them by telephone to discuss whether they would be able to discuss their experiences. If their response is favourable then a separate consent form will be posted to them and completed prior to being invited to attend an individual interview at their convenience. The interviews may be conducted at the patients’ home or physiotherapy department.

The eligible physiotherapists will be approached as a group during the regular training sessions held throughout the duration of the trial, led by the chief investigator, and an open letter including a participant information sheet and consent form will invite them to contact CL with a view to discussing participation in this aspect of the study. If the physiotherapist wishes to participate then a mutually convenient individual interview at the physiotherapy department will be scheduled.

It is expected that interviews will last between 30 to 60 minutes. Purposive sampling will continue until data saturation. Data saturation is the point where on-going analysis reveals no new themes emerging from the data but it is estimated that 10 to 20 patient interviews will be required and up to 10 therapist interviews. Once it is thought that data saturation has been attained, 2 more interviews will be conducted to confirm this.

### Data collection

Interviews will be directed by topic guides These discussions will be recorded using a digital voice recorder and subsequently transcribed verbatim.

### Data analysis

CL and SM will analyse the data using the framework approach.

The framework approach has been developed specifically for applied research in which the objectives of the investigation are set *a* priori [33]. The 5 stages of data analysis associated with the framework approach are as follows:

1. Familiarisation – identifying key ideas and themes

2. Identifying a thematic framework – identifying all key issues, concepts and themes by which the data can be examined

3. Indexing – application of the thematic framework to all the data

4. Charting – Organisation of the data according to the defined thematic framework to which they relate to form common charts

5. Mapping and Interpretation – using the charts to define concepts, map the range and nature of phenomena, create typologies and find associations with a view to providing explanations for the findings.

One clear advantage of the framework method is the systematic and visible stages of the analysis process [34,35]. The patients and physiotherapists involved will be invited to inspect the outcomes of the analysis in an attempt to maximise validity of the interpretations.

### Patient & public involvement

A formal patient and public involvement event was held with the aim of seeking lay opinion regarding the design and conduct of the SELF study. A focus group led by CL and JA was undertaken with a sample of volunteers currently attending for physiotherapy at Doncaster & Bassetlaw Hospitals NHS Foundation Trust. In summary, the lay group found our initial proposals acceptable but suggested that initial approach by letter, a full description of the content of the treatment arms, an enhanced exercise diary incorporating a visual illustration of any prescribed exercise and encouragement to the physiotherapists involved to help set specific treatment goals might improve the design and conduct of the SELF study. These features have been incorporated into this current version of the protocol.

## Ethical approval

The protocol was approved by the National Research Ethics Service Committee Yorkshire & the Humber – Leeds West, UK on the 6^th^ January 2012.

## Competing interests

The authors declare that they have no competing interests.

## Authors’ contributions

CL was responsible for the conception of the idea and drafting of the initial manuscript. All authors were involved in the design, revisions to the manuscript and final approval of the version to be published.

## Authors’ information

This work is supported by a National Institute for Health Research (NIHR) Doctoral Research Fellowship (DRF) awarded to CL. SW, SM and SMay are academic supervisors and JA is a clinical partner.

## Pre-publication history

The pre-publication history for this paper can be accessed here:

http://www.biomedcentral.com/1471-2474/13/62/prepub
